# A Culture-Proven Case of Community-Acquired *Legionella* Pneumonia Apparently Classified as Nosocomial: Diagnostic and Public Health Implications

**DOI:** 10.1155/2013/303712

**Published:** 2013-02-11

**Authors:** Annalisa Bargellini, Isabella Marchesi, Patrizia Marchegiano, Luca Richeldi, Roberto Cagarelli, Greta Ferranti, Paola Borella

**Affiliations:** ^1^Department of Clinical, Diagnostic and Public Health Medicine, University of Modena and Reggio Emilia, Via Campi 287, 41125 Modena, Italy; ^2^Prevention and Protection Service, Health Direction, University Hospital, Via Del Pozzo 71, 41124 Modena, Italy; ^3^Department of Medical and Surgical Sciences for Children and Adults, University of Modena and Reggio Emilia, Via Del Pozzo 71, 41124 Modena, Italy; ^4^Public Health Authority, Emilia Romagna Region, Via Aldo Moro 21, 40127 Bologna, Italy

## Abstract

We report a case of *Legionella* pneumonia in a 78-year-old patient affected by cerebellar haemangioblastoma continuously hospitalised for 24 days prior to the onset of overt symptoms. According to the established case definition, this woman should have been definitely classified as a nosocomial case (patient spending all of the ten days in hospital before onset of symptoms). Water samples from the oncology ward were negative, notably the patient's room and the oxygen bubbler, and the revision of the case history induced us to verify possible contamination in water samples collected at home. We found that the clinical strain had identical rep-PCR fingerprint of *L. pneumophila* serogroup 1 isolated at home. The description of this culture-proven case of Legionnaires' disease has major clinical, legal, and public health consequences as the complexity of hospitalised patients poses limitations to the rule-of-thumb surveillance definition of nosocomial pneumonia based on 2–10-day incubation period.

## 1. Introduction

*L. pneumophila* is one of the leading causes of community and hospital-acquired pneumonia, the latter having a higher fatality rate [1]. It is therefore important to identify the clinical characteristics and radiographic findings rapidly. 

The disease has no particular clinical features that clearly distinguish it from other types of pneumonia [2], although progression of pulmonary infiltrates despite appropriate antibiotic therapy might be suggestive of Legionnaires' disease [3]. The suspicion of Legionnaires' disease should arise from an adequate epidemiologic and clinical context, but confirmation requires specific diagnostic tests: urinary antigen detection and PCR are more rapid than culture and/or seroconversion, although culture combined with molecular typing remains the gold standard [4].

The availability of the clinical strain is essential to identify the environmental source of infection, that is not always the most expectable [5]. In a cluster among residents of a long-term care facility, no contamination was detected within the structure, but the clinical *L. pneumophila *strain was found similar to that isolated from an industrial cooling tower. The authors suggested that *Legionella* entered the structure through the air-intake system; therefore, the association between a case and the source of infection should not be taken for granted [6].

We here describe a culture-confirmed case of community-acquired *Legionella* pneumonia in a patient continuously hospitalised for 24 days. Because of the complexity of the clinical picture of hospitalised patients, special attention was devoted to identify the origin of infection correctly, according to the nosocomial-acquired case definition of Legionnaires' disease.

## 2. Case Description

A 78-year-old woman was admitted to the Internal Medicine ward of our university hospital on October 8, 2009, for a probable left hemispheric ischemia, difficulty in deambulation and speech, and signs of pseudobulbar encephalopathy. She was afebrile and both chest radiogram and CT scan were unremarkable. Brain CT and MRI showed cerebellar mass compatible with primary neoplasm; dexamethasone (24 mg/die) was started and she was discharged for amelioration of symptoms on November 12. One week later, the patient was readmitted to the oncology ward, based on a Von Hippel-Lindau syndrome (cerebellar haemangioblastoma).

Discharged again on December 1, she stayed at home for 3 weeks until December 21, when she was readmitted to the oncology ward for the worsening of neurological symptoms and a concomitant mild dyspnoea. The patient was afebrile, conscious, and collaborative, without deficit in strength or sensitivity in both arms and legs. A chest X-ray did not show abnormal findings. During hospital admission, on January 13, the patient became febrile, hypoxemic, and hypercapnic, and did undergo both chest CT scan and bronchoscopy with bronchoalveolar lavage (BAL). The presence of a parenchymal consolidation in the right lower lung prompted the diagnosis of pneumonia. The patient was transferred to the ICU, treated with wide-spectrum antibiotics and noninvasive ventilation. The day after, the urinary antigen test for *Legionella* (Biotest urinary antigen EIA, Germany) was positive and levofloxacin was started. Other urinary samples collected between January 18 to February 1 tested positive for *Legionella,* and *L. pneumophila* serogroup 1 was identified by latex agglutination test (Oxoid, UK) on the BAL sample. The BAL fluid also tested positive for *Haemophilus influenzae* and cytomegalovirus, whereas the pharyngotonsillar exudate was positive for *Candida albicans*. Anti-legionella antibodies (Serion ELISA; Institut Virion∖SerionGmbH, Wurzburg, Germany) were detectable on a serum sample collected on January 20, whereas the serum sample stored from the October 26 was negative. The clinical conditions of the patient improved and the chest X ray performed on February 3 was normal. On February 22, the patient died in the hospital due to a rapid worsening of cerebellar tumour with a concomitant aspiration pneumonia.

## 3. Environmental Investigation

Since 1999, a surveillance programme has been maintained in the hospital to assess the environmental contamination of *Legionella *spp. in hot water distribution systems [7]. According to national guidelines which require adoption of control measures when *Legionella* contamination exceeds 10^4^ CFU/L [8], a continuous chlorine dioxide system was installed on June 2009 in the oncology network due to high levels of contamination found. Following the case occurrence, water samples from the oncology ward were immediately collected for *Legionella* analysis, including the patients' rooms and the oxygen bubbler. All points were negative except for the tank of the hot water distribution system that was contaminated by *L. pneumophila* serogroup 1 (2100 CFU/L). These results and the revision of the case history induced us to verify if *Legionella* infection was present at the re-admission time; thus, water samples were collected at home where the patient stayed from December 1 to December 21. *L. pneumophila* serogroup 1 was detected in the patient's home shower (1400 CFU/L without flushing and 50 CFU/L after flushing) and in the tank of the patient's home central distribution system (7500 CFU/L). 

**Figure 1:**
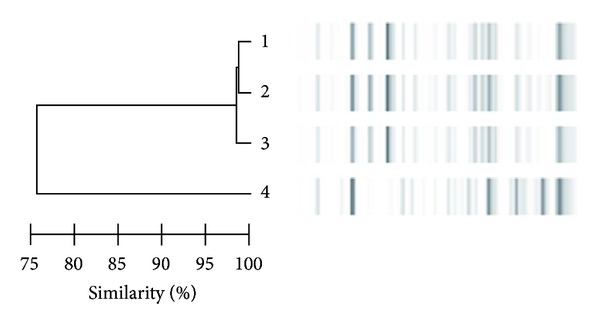
Rep-PCR analysis: dendrogram of similarity and molecular profiles of *L. pneumophila* serogroup 1 isolated from various specimens. (1) Isolates from home shower without flushing. (2) Isolates from home shower after flushing. (3) Clinical strain. (4) Isolates from the tank of the oncology ward.

Repetitive element-polymerase chain reaction (rep-PCR) [9] was used to compare clinical and environmental *L. pneumophila *isolates. The clinical strain and only the ones isolated from home shower showed identical restriction pattern (Figure 1). The national reference laboratory (Istituto Superiore di Sanità) confirmed the similarity, by using monoclonal antibodies and analysis of genomic pattern by amplified fragment length polymorphism, and established that the clinical and home isolates of *L. pneumophila* serogroup 1 belonged to the Knoxville strain.

## 4. Discussion and Conclusions

The case described supports the concept that critical patients with *Legionella* pneumonia can come to clinical observation with nonspecific clinical and radiological presentation, developing a full-blown clinical picture after a long period. These cases may be categorized as nosocomial according to the clinical definition of Legionnaires' disease (“patients who spent all of the ten days in hospital before onset of symptoms”) [10], while being community acquired. In this patient who was continuously hospitalised for 24 days, the clinical strain had identical rep-PCR fingerprint of *L*. *pneumophila* serogroup 1 isolated from her home shower and was different from the strains isolated in the hospital.

We hypothesize that the use of a low dose of dexamethasone for two months could have had a role in camouflaging the ongoing infection, thus favouring a subtle and insidious appearance of clinical symptoms which were not accompanied by radiological evidence. Mild dyspnoea present at hospital admission cannot be considered as specific and might be due to other concomitant causes, namely, chronic use of systemic steroids and chronic heart failure. Screening with *Legionella* urinary test all patients admitted to the hospital with such mild symptoms is impracticable and uneconomic. An appropriate diagnostic management is instead mandatory after the clear appearance of clinical signs of pneumonia to avoid fatality risk. To confirm this, our case was rapidly identified thanks to the surveillance protocol, which includes the contemporary collection of serum, urine, and secretions or other biological specimens on all patients exhibiting pneumonia [11]. Screening with urinary test and BAL fluid culture were essential to select the appropriate antibiotic therapy, substituting the wide-spectrum one probably not effective in the presence of a *Legionella *infection.

The description of this culture-proven case of Legionnaires' disease highlights the limitations of the rule-of-thumb surveillance definition of nosocomial pneumonia based on the usual incubation period for *Legionella* of 2–10 days. In a large outbreak of *Legionella *pneumonia in The Netherlands, the reported incubation period was 2 to 19 days (median 7 days) [12], and outliers up to 26 days have been described [13]. In these situations, the conduction of an appropriate genetic correlation between the index case and the potential environmental source is the only procedure able to assign the source of infection correctly [14, 15]. Actually, the presence of *L. pneumophila *serogroup 1 in both tank of the oncology network and residential setting did not allow an immediate comprehension of the infection source, and only the comparison of clones with molecular methods solved the diagnostic puzzle. 

We are conscious that resources will not likely be available for environmental microbiologic investigation for the purpose of improving the accuracy of designating case as community versus nosocomial-acquired pneumonia. However, our case report may help to avoid the taken-for-granted association between hospitals and all cases occurred 10 days after recovery, with the consequent negative publicity in the newspaper and television and/or lawsuits based on allegations of negligence [16].

Lastly, we consider it relevant to inform patients undergoing long-term immunosuppressive therapy and/or affected by chronic degenerative disorders to be at higher risk for severe *Legionella* infection when returning home. They should pay attention to aerosolized water, to reduce shower exposure in any community setting, and, eventually, to test for *Legionella *spp. the domestic water supply [17].

## References

[1] Dominguez A, Alvarez J, Sabria M (2009). Factors influencing the case-fatality rate of Legionnaires’ disease. *International Journal of Tuberculosis and Lung Disease*.

[2] Gupta SK, Imperiale TF, Sarosi GA (2001). Evaluation of the Winthrop-University Hospital criteria to identify Legionella pneumonia. *Chest*.

[3] Diederen BMW (2008). Legionella spp. and Legionnaires’ disease. *Journal of Infection*.

[4] Carratalà J, Garcia-Vidal C (2010). An update on Legionella. *Current Opinion in Infectious Diseases*.

[5] Borella P, Marchesi I, Boccia S (2006). Epidemiological investigation on a suggestive case of Legionella pneumonia and public health implications. *Scandinavian Journal of Infectious Diseases*.

[6] Phares CR, Russell E, Thigpen MC (2007). Legionnaires’ disease among residents of a long-term care facility: the sentinel event in a community outbreak. *American Journal of Infection Control*.

[7] Marchesi I, Marchegiano P, Bargellini A (2011). Effectiveness of different methods to control legionella in the water supply: ten-year experience in an Italian university hospital. *Journal of Hospital Infection*.

[8] (2000). Italian guidelines for prevention and control of legionellosis. *Gazzetta Ufficiale Della Repubblica Italiana, Serie Generale*.

[9] Haroon A, Koide M, Higa F, Hibiya K, Tateyama M, Fujita J (2010). Repetitive element-polymerase chain reaction for genotyping of clinical and environmental isolates of Legionella spp. *Diagnostic Microbiology and Infectious Disease*.

[10] World Health Organization (2007). *Legionella and the Prevention of Legionellosis*.

[11] Borella P, Boccia S, Leoni E, Cianciotto NP, Abu Kwaik Y, Edelstein PH (2006). Prevalence of Legionnaires' disease and investigation on risk factors: results on an Italian multicentric study. *Legionella: State of the Art 30 Years after Its Recognition*.

[12] den Boer JW, Yzerman EPF, Schellekens J (2002). A large outbreak of Legionnaires’ disease at a flower show, the Netherlands, 1999. *Emerging Infectious Diseases*.

[13] Fraser DW, Tsai TR, Orenstein W (1977). Legionnaires’ disease. Description of an epidemic of pneumonia. *New England Journal of Medicine*.

[14] Drenning SD, Stout JE, Joly JR, Yu VL (2001). Unexpected similarity of pulsed-field gel electrophoresis patterns of unrelated clinical isolates of Legionella pneumophila, serogroup 1. *Journal of Infectious Diseases*.

[15] Thouverez M, Godard C, Leprat R, Talon D (2003). Is pulsed-field gel electrophoresis a valuable tool to identify nosocomial cases of Legionella pneumophila disease?. *Journal of Hospital Infection*.

[16] Stout JE, Yu VL (2010). Environmental culturing for Legionella: can we build a better mouse trap?. *American Journal of Infection Control*.

[17] Sax H, Dharan S, Pittet D (2002). Legionnaires’ disease in a renal transplant recipient: nosocomial or home-grown?. *Transplantation*.

